# A Radiological Evaluation of Lumbar Spinous Processes and Interspinous Spaces, Including Clinical Implications

**DOI:** 10.7759/cureus.19454

**Published:** 2021-11-10

**Authors:** Dicle Kaya Ayvaz, Piraye Kervancıoğlu, Ayşe Bahşi, İlhan Bahşi

**Affiliations:** 1 Department of Anatomy, Gaziantep University, Gaziantep, TUR; 2 Clinic of Physical Medicine and Rehabilitation, Dr. Ersin Arslan Education and Research Hospital, Gaziantep, TUR

**Keywords:** spine, lumbar vertebrae, lumbar interspinous spacer, morphometry, interspinous space, spinous process

## Abstract

Background and objective

The aim of this study was the examination of morphometry of the spinous process (SP) and interspinous space (ISS) of the lumbar region to help provide a basis for the design and implantation of interspinous devices.

Methods

Between 2017 and 2019, 215 individuals underwent magnetic resonance imaging of the lumbar region for various reasons. No pathology was detected in these images, and the participants' age, height, and weight information when available were included in the study. From these images, the height and length of the SP and ISS in the lumbar region were noted. The heights of the SP and ISS were measured at three levels as anterior, middle, and posterior (respectively, anterior height of the spinous process* *[AHSP], middle height of the spinous process [MHSP], as well as posterior height of the spinous process [PHSP] for the height of SP, and anterior ISS, middle ISS and posterior ISS for the height of ISS). All measurements were compared according to the gender, age, weight, height, and body mass index of the individuals.

Results

The level with the lowest SP height and length was L5 vertebra. The ISS height and length were lowest at L4-L5. In addition, we observed a statistically significant difference at multiple levels with age, weight, height, and body mass index of the reference ranges.

Conclusion

We think that these changes should be considered when designing and implanting interspinous devices. Since there are few studies examining all these correlations, we think that the results of this study will make a unique contribution to the literature.

## Introduction

Lumbal spinal stenosis (LSS) is a clinical syndrome resulting from degenerative changes in the lumbar region [1]. Degeneration in the lumbar region may cause narrowing of the vertebral canal and intervertebral foramen, as well as compression of the spinal nerves [2, 3]. The most noticeable characteristics of intermittent neurogenic claudication in patients with LSS are pain, weakness and numbness in the legs [4]. LSS patients are usually asymptomatic when they are sitting, with symptoms appearing while standing or walking [5].

It has been reported that LSS is associated with aging populations and is one of the prime reasons for spinal surgery in individuals over the age of 65 [6]. There are both conservative and surgical treatment options for symptomatic LSS patients [7]. On the other hand, spinal surgery procedures are evolving towards less invasive techniques [8]. Moreover, interspinous devices are an important alternative as they are less invasive than traditional surgical techniques [9]. Therefore, there has been a burgeoning interest in interspinous devices for the treatment of LSS in recent years [10].

Although implanting interspinous devices in patients is advantageous when compared to traditional surgical techniques, studies report that more studies are needed to clarify whether this method is a safe and effective alternative [3, 9, 11]. Common complications with implanting interspinous devices include slipping of the device from the ISS if the implanted interspinous device is too small, or fracture of the SP if it is too large [8, 12, 13]. The morphometric values of SP and ISS should be known in order to design and implant interspinous devices in appropriate sizes [4, 14, 15]. It has been reported that the morphometric values of SP and ISS correlate with sex [14-17], age [16-18], weight and height [4].

We think that the morphometric values of SP and ISS and the relationship of these values with sex, age, weight, height, and body mass index (BMI) should be known in order to allow for the successful implantation of interspinous devices and mitigate complications. For this reason, we aimed to examine the morphometry of the SP and ISS of the lumbar region to help provide a basis for the design and implantation of interspinous devices.

## Materials and methods

Data collection

This retrospective study was approved by the Ethics Committee of Gaziantep University (Approval date: 19/06/2019, number: 231). Between 2017 and 2019, 215 individuals (male, 100; female, 115) who reported to the Physical Medicine and Rehabilitation Clinic of Dr. Ersin Arslan Training and Research Hospital and underwent magnetic resonance imaging of the lumbar region for various reasons were included in the study. No pathology was detected in these images, and the age, height, and weight information that were available were included in the study. All images with a slice thickness of 4 mm were obtained using GE Signa Excite HDI 1.5 Tesla (GE Healthcare, Chicago, IL).

Inclusion and exclusion criteria

Images from patients aged 18-65 years were included in the study. Patients with images containing artifacts that could lead investigators to an incomplete or incorrect conclusion and prevent the determination of reference points were excluded from the study. Moreover, patients with scoliosis, lumbarization, socialization, and bone deformities were not included in the study.

Examiner reliability

All measurements were taken by a single person. One month after the measurements were taken, 10% of the cases were re-examined by the same investigator with the names of the patients hidden, and no differences were found that deviated from the first round of measurements (p>0.05).

Measurement of morphometric parameters

Images were analyzed using RadiAnt DICOM Viewer 2020.1.1 Software. In the sagittal sections of the images, SPs of five vertebrae, from the first lumbar vertebra (L1) to the fifth lumbar vertebra (L5) as well as four ISSs between these SPs were examined.

Parameters examined

**Figure 1:**
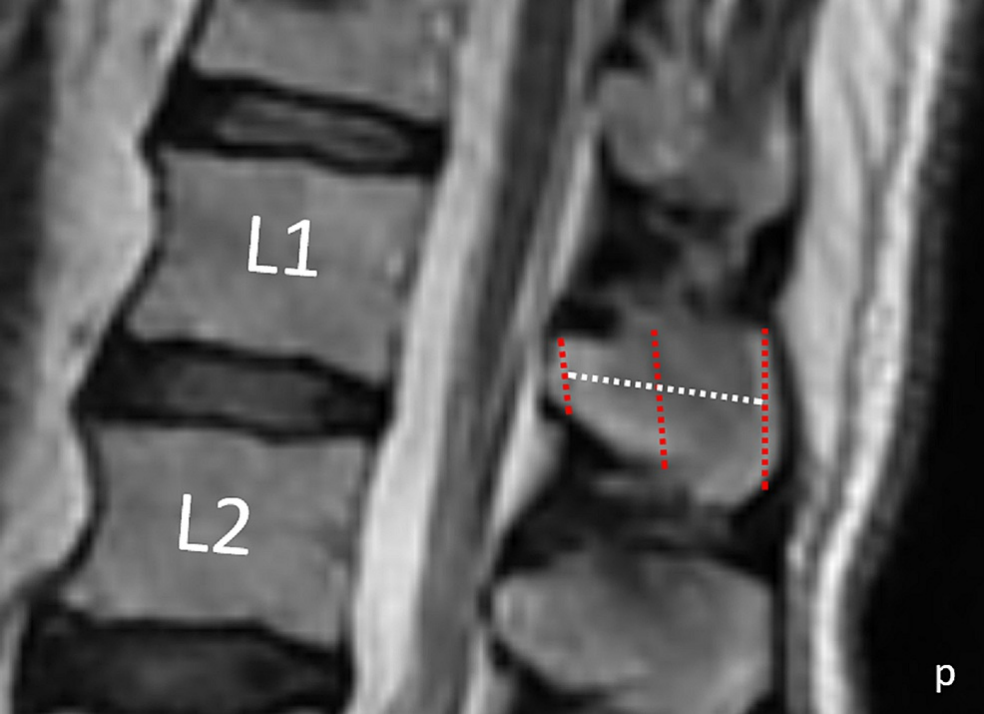
Measurement of height and length of the SP The red lines are AHSP, MHSP, and PHSP in order of anterior to the posterior, the white line is LSP, and 'p' indicates posterior. Abbreviations: SP=spinous process; AHSP=anterior height of the spinous process; MHSP=middle height of the spinous process; PHSP=posterior height of the spinous process; LSP=Length of the spinous process

The following parameters were examined: the posterior height of the spinous process (PHSP), the distance between the rear-top and rear-bottom points of the SP; anterior height of the spinous process (AHSP), the distance between the most anterior-upper point of the SP and the point where the ligamentum flavum gets cambered in the anterior-bottom part of the SP; the length of the spinous process (LSP), the distance between midpoints of PHSP and AHSP; and middle height of the spinous process (MHSP): the upper reference points of the SP at the front and rear were joined by a line, as were the lower reference points at the front and back; the midpoints of these lines were determined, as was the line in the superior-inferior direction of these midpoints. The distance between the top and bottom points of SP on this line was MHSP (Figure 1).

**Figure 2:**
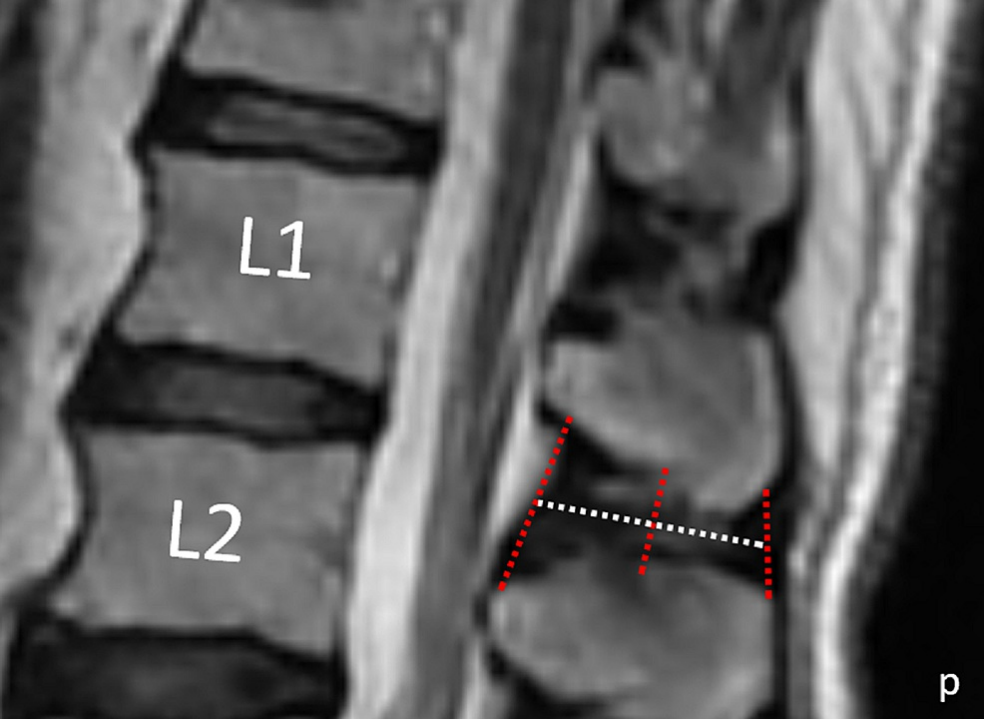
Measurement of height and length of the ISS The red lines are AHISS, MHISS, and PHISS in order of anterior to the posterior, the white line is LISS, and 'p' indicates posterior. Abbreviations: ISS=Interspinous space; AHISS=anterior height of the interspinous space; MHISS=middle height of the interspinous space; PHISS=posterior height of the interspinous space; LISS=Length of the interspinous space

Other parameters examined include the posterior height of the interspinous space (PHISS): the distance between the lower point of the PHSP above and the upper point of the PHSP below; the anterior height of the interspinous distance (AHISS): the distance between the lower point of the AHSP above and the upper point of the AHSP below; length of the interspinous distance (LISS): Distance between midpoints of AHISS and PHISS; and the middle height of the interspinous distance (MHISS): the posterior-lower and anterior-lower points of the SP above it were joined by a line, as were the posterior-upper and anterior-upper points of the SP below it. The midpoints of these lines were determined. as was the line in the superior-inferior direction of these midpoints. The distance between the top and bottom points of ISS on this line was MHISS (Figure 2).

Statistical analysis

For normally distributed values, in accordance with the Shapiro Wilk test, Student's t-test was used for the comparison of two groups. Relationships between numerical variables were determined using the Pearson correlation coefficient. Mean ± standard deviation for numerical variables, as well as quantity values for categorical variables, were given as descriptive statistics. SPSS Statistics for Windows v. 22.0 (IBM, Armonk, NY) was used for statistical analysis, and p<0.05 was accepted as statistically significant.

## Results

In this study, MRI images of 100 males (mean age: 40.28±11.29) and 115 females (mean age: 42.53±9.09) aged 18-65 years were examined. There was no statistically significant difference between age and gender (p=0.107). The age, height, weight and BMI values of the subjects and the comparison of these values by sex are given in Table 1. The mean height was statistically and significantly higher among male patients while the same was true for average BMI values in female patients (p=0.001, p=0.001, respectively).

**Table 1:** Results of the age, weight, height and BMI *Statistically significant difference (p<0.05) M: male; F: female; T: total, N: number of individuals; SD: standard deviation

Parameter	M		F		T		p
	N	Mean±SD	N	Mean±SD	N	Mean±SD	
Age	100	40.28±11.29	115	42.53±9.09	215	41.48±10.21	0.107
Weight (kg)	100	78.64±14.21	115	76.09±13.46	215	77.27±13.84	0.178
Height (cm)	100	170.69±6.93	115	160.03±5.53	215	165.00±8.18	0.001*
BMI (kg/m²)	100	26.98±4.56	115	29.71±5.03	115	28.44±5.00	0.001*

Values for parameters measured in SPs are given in Table 2. The correlation of the parameters measured in SPs with age, weight, height, and BMI is shown in Table 3.

**Table 2:** Results of morphometric data of the SPs M: male, F: female, T: total, N: number of individuals, SD: standard deviation *statistically significant difference: p<0.05

Level	Parameter	M		F		T		p
		N	Mean±SD	N	Mean±SD	N	Mean±SD	
L1	PHSP (mm)	24	17.47±3.55	21	15.81±2.44	45	16.70±3.16	0.078
	MHSP (mm)	24	16.96±3.33	21	16.00±2.85	45	16.51±3.12	0.305
	AHSP (mm)	24	10.09±2.38	21	9.60±1.78	45	9.86±2.11	0.445
	LSP (mm)	24	23.74±3.21	21	19.97±2.88	45	21.98±3.58	0.001*
L2	PHSP (mm)	34	18.88±2.49	40	18.16±2.94	74	18.49±2.75	0.260
	MHSP (mm)	34	18.05±3.01	40	17.76±3.11	74	17.89±3.05	0.689
	AHSP (mm)	34	10.36±2.32	40	9.29±1.36	74	9.78±1.92	0.017*
	LSP (mm)	34	26.33±2.90	40	22.89±2.60	74	24.47±3.22	0.001*
L3	PHSP (mm)	46	17.52±3.39	57	16.36±3.27	103	16.88±3.36	0.082
	MHSP (mm)	46	20.52±3.47	57	19.65±3.52	103	20.04±3.51	0.211
	AHSP (mm)	46	11.35±2.96	57	9.35±1.80	103	10.25±2.58	0.001*
	LSP (mm)	46	25.89±5.11	57	24.38±3.38	103	25.05±4.28	0.073
L4	PHSP (mm)	64	14.88±3.21	90	13.50±2.90	154	14.07±3.10	0.006*
	MHSP (mm)	64	18.91±3.27	90	17.80±2.81	154	18.26±3.05	0.025*
	AHSP (mm)	64	11.68±4.39	90	8.94±2.37	154	10.08±3.61	0.001*
	LSP (mm)	64	24.92±3.22	90	22.78±3.38	154	23.67±3.47	0.001*
L5	PHSP (mm)	30	11.63±2.75	35	11.19±2.88	65	11.39±2.80	0.528
	MHSP (mm)	30	14.51±2.96	35	13.72±2.83	65	14.08±2.89	0.277
	AHSP (mm)	30	10.20±3.16	35	8.66±2.62	65	9.37±2.96	0.035*
	LSP (mm)	30	22.81±3.69	35	21.26±3.35	65	21.97±3.57	0.080

**Table 3:** Correlation of the age, weight, height and BMI with each parameter of the SP *statistically significant difference

Level	Parameter	Age		Weight		Height		BMI	
		p	r	p	r	p	r	p	r
L1	PHSP	0.112	-	0.773	-	0.018*	0.350	0.083	-
	MHSP	0.759	-	0.581	-	0.543	-	0.408	-
	AHSP	0.201	-	0.032*	-0.319	0.996	-	0.018*	-0.350
	LSP	0.677	-	0.001*	0.473	0.008*	0.389	0.064	-
L2	PHSP	0.665	-	0.861	-	0.045*	0.234	0.407	-
	MHSP	0.062	-	0.875	-	0.535	-	0.910	-
	AHSP	0.199	-	0.532	-	0.242	-	0.203	-
	LSP	0.477	-	0.030*	0.253	0.001*	0.435	0.645	-
L3	PHSP	0.036*	-0.207	0.772	-	0.024*	0.223	0.089	-
	MHSP	0.733	-	0.108	-	0.162	-	0.018*	-0.232
	AHSP	0.838	-	0.751	-	0.001*	0.323	0.019*	-0.231
	LSP	0.001*	0.310	0.366	-	0.049*	0.194	0.914	-
L4	PHSP	0.605	-	0.206	-	0.010*	0.206	0.006*	-0.220
	MHSP	0.319	-	0.314	-	0.012*	0.202	0.019*	-0.189
	AHSP	0.284	-	0.811	-	0.001*	0.292	0.063	-
	LSP	0.210	-	0.194	-	0.001*	0.299	0.562	-
L5	PHSP	0.705	-	0.045*	0.250	0.963	-	0.069	-
	MHSP	0.498	-	0.361	-	0.050	-	0.808	-
	AHSP	0.215	-	0.046*	-0.248	0.161	-	0.008*	-0.328
	LSP	0.021*	0.286	0.086	-	0.401	-	0.210	-

Values for parameters measured in ISSs are given in Table 4. The correlation of parameters measured in ISSs with age, weight, height and BMI is shown in Table 5.

**Table 4:** Results of morphometric data of the ISSs M: male; F: female; T: total; N: number of individuals; SD: Standard deviation All measurement units are given in mm. *statistically significant difference

Level	Parameter (mm)	M		F		T		p
		N	Mean±SD	N	Mean±SD	N	Mean±SD	
L1-l2	PHISS	33	14.88±3.21	39	13.56±2.66	72	14.17±2.98	0.060
	MHISS	33	14.38±4.32	39	12.91±3.77	72	13.58±4.07	0.129
	AHISS	33	20.57±4.29	39	20.61±3.45	72	20.59±3.83	0.963
	LISS	33	24.60±3.46	39	21.91±3.17	72	23.14±3.55	0.001*
L2-L3	PHISS	45	14.13±3.17	53	12.99±3.29	98	13.52±3.27	0.086
	MHISS	45	12.65±3.81	53	10.94±2.99	98	11.73±3.48	0.015*
	AHISS	45	21.31±3.61	53	21.09±2.61	98	21.19±3.10	0.737
	LISS	45	26.46±3.73	53	24.00±3.65	98	25.13±3.87	0.001*
L3-L4	PHISS	61	12.44±3.08	91	10.90±2.88	152	11.52±3.05	0.002*
	MHISS	61	9.12±2.70	91	8.63±7.64	152	8.83±6.14	0.635
	AHISS	61	19.27±3.91	91	18.73±3.51	152	18.95±3.67	0.381
	LISS	61	25.48±3.64	91	23.58±3.26	152	24.34±3.54	0.001*
L4-L5	PHISS	53	10.61±2.58	71	8.70±1.89	124	9.52±2.40	0.001*
	MHISS	53	7.40±1.62	71	6.79±1.31	124	7.05±1.48	0.023*
	AHISS	53	16.32±3.71	71	16.49±2.87	124	16.42±3.24	0.774
	LISS	53	23.52±3.53	71	21.60±3.55	124	22.42±3.65	0.003*

**Table 5:** Correlation of the age, weight, height and BMI with each parameter of the ISSs *statistically significant difference

Level	Parameter	Age		Weight		Height		BMI	
		p	r	p	r	p	r	p	r
L1-L2	PHISS	0.127	-	0.184	-	0.001*	0.371	0.799	-
	MHISS	0.014*	-0.287	0.561	-	0.012*	0.295	0.342	-
	AHISS	0.135	-	0.179	-	0.671	-	0.351	-
	LISS	0.929	-	0.010*	0.300	0.001*	0.391	0.241	-
L2-L3	PHISS	0.888	-	0.207	-	0.008*	0.267	0.566	-
	MHISS	0.021*	-0.233	0.346	-	0.001*	0.318	0.175	-
	AHISS	0.081	-	0.103	-	0.061	-	0.743	-
	LISS	0.008*	0.268	0.023*	0.230	0.001*	0.360	0.799	-
L3-L4	PHISS	0.620	-	0.541	-	0.028*	0.178	0.544	-
	MHISS	0.099	-	0.409	-	0.121	-	0.074	-
	AHISS	0.398	-	0.145	-	0.200	-	0.029*	-0.177
	LISS	0.146	-	0.140	-	0.001*	0.311	0.641	-
L4-L5	PHISS	0.176	-	0.139	-	0.001*	0.299	0.654	-
	MHISS	0.267	-	0.117	-	0.011*	0.226	0.979	-
	AHISS	0.481	-	0.225	-	0.262	-	0.582	-
	LISS	0.879	-	0.432	-	0.017*	0.213	0.680	-

## Discussion

The main purpose of treating LSS is to maintain or improve the biomechanical function of the lumbar region by largely preserving its anatomical structure. If adequate neural decompression is achieved through treatment, symptoms can be relieved [19]. Conservative treatment can be tested to reduce LSS symptoms [20]. For patients who do not respond to conservative treatment and are showing progressive symptoms, surgical techniques are considered [7, 20]. Complications caused by surgical techniques have moved surgeons towards more minimally invasive techniques [3]. The implantation of interspinous devices is a minimally invasive option for spinal surgery [2, 11, 12].

Lee et al. [21] stated that implantation of interspinous devices is an alternative for patients, and is as risk-free as conservative treatments and as effective as surgical treatments for patients, especially elderly ones who cannot withstand surgical complications 21]. For this reason, patients who do not respond to conservative treatment can have this procedure done before any surgical intervention is carried out [2]. While interspinous devices allow for flexion, lateral flexion, and rotation [22], they restrict extension [12], reduce strain on the intervertebral discs and zygapophysial joints [23], and improve stenosis in the intervertebral foramen through distraction [12, 23].

Besides all these advantages, when implanting an interspinous device, it is important to ensure the correct device size and choose the right patient [9, 12]. Attention should also be paid to the bone density of patients undergoing the procedure [12]. There is a need for more information about the morphometry of SP and ISS so that complications such as SP fractures or slipping of the interspinous device due to incorrect device size can be eliminated, and implanted devices can function more safely and effectively [4, 14, 15].

Morphometric comparison of SP measurements

SPs protect neural structures in the vertebral canal and serve as a foothold for interspinous ligaments, supraspinous ligaments, and intersegmental paraspinal muscles. Therefore, they have an important anatomical and biomechanical function. Furthermore, SPs are clinically important as they affect access to the vertebral canal during decompressive surgical interventions. Significant changes occur in the morphology of SPs with increasing age. It has been reported that these changes may affect surgical methods and spinal injection techniques, and should be considered in the design and implantation of interspinous devices [17]. We also think that the dimensions of SPs and their correlation with sex, age, weight, height, and BMI are important in the design and implantation of interspinous devices. To allow for a more comprehensive comparison in our study, we measured the height of the SPs in their anterior, middle, and posterior locations.

Several studies reported that the SP as being at its lowest height at the L5 vertebra, among the lumbar vertebrae [4, 15, 17, 24]. This study also demonstrated similar results. On the other hand, while Ihm et al. [18] found SP to be at its highest at L2 vertebra, other studies found that it was highest at L3 vertebra [16, 24]. In a study by Lin et al. [16], in which they take height measurements in the anterior, middle, and posterior locations using the same measurement method as this study, PHSP was reported as being at its highest at L2, while AHSP and MHSP were at their highest at L3, in line with the findings of this study.

Lin et al. [16] and Aylott et al. [17] report that males have higher SP heights than females. Similarly, this study found that AHSP was higher in males than females at the L2, L3, and L4 vertebrae, PHSP was higher in L4 vertebra.

Aylott et al. [17] state that there is a positive correlation between SP height and age. Paholpak et al. [25] and Shaw et al. [15] found that the height of L4 and L5 SPs increase with age. Contrary to these studies, in this study, it was found a negative correlation between the PHSP of L3 vertebra and age. It was found that there was no correlation at other levels.

Similar to the findings in this study, some studies report that the lowest LSP value is at the L5 vertebra [4, 15, 24]. Other studies report the highest LSP value at the L3 vertebra [16, 18, 24]. Cai et al. [14] found the highest LSP value at L3 vertebra in males and L4 vertebra in females. In this study, the highest LSP value was measured at L2 vertebra for males; at L3 vertebra for females.

It has been reported that LSP is higher in men than in women in all lumbar vertebrae [14-16]. This study found that LSP was higher in males than in females at L1, L2, and L4 vertebrae in accordance with the literature.

Ruhli et al. [26] argue that LSP does not change with age. Contrary to Ruhli et al. [26] in this study it was found a positive correlation between LSP and age at L3 and L5 vertebrae.

Morphometric comparison of ISS measurements

If the ISS is larger than the device to be implanted, the interspinous device remains loose and cannot provide the necessary distraction. If it is small, the risk of the SP fracture increases. The height and length of the ISS are therefore important for the successful implantation of an interspinous device [8]. To allow for a more comprehensive comparison in our study, we measured the height of the ISS in its anterior, middle, and posterior parts.

Ihm et al. [18] found that ISS was at its highest at the L2-L3. Leng et al. [24] measured the highest PHISS and AHISS value at L2-L3, and the highest MHISS value at L1-L2. Similarly, in this study, AHISS was at its largest at the L2-L3 level, while PHISS and MHISS were largest at the L1-L2. Moreover, it has been reported that the L4-L5 segment, where most surgeries take place, has the lowest height [4]. In line with the literature, in this study, PHISS, MHISS, and AHISS were at their lowest at L4-L5.

Cai et al. [14] state that there is no statistically significant difference between genders in terms of height measurements of the ISS. On the other hand, Sobottke et al. [4] and Lin et al. [16] report that the ISS height is greater in men than in women. Sobottke et al. [4] found that this difference was only present in ISS at L3-L4. In line with the literature, in this study, PHISS was higher in males than females at L3-L4 and L4-L5, and MHISS was higher at L2-L3 and L4-L5. As reported in Lin et al. [16], we believe that sex differences should be taken into account in the design and implantation of the interspinous device, since the height of the ISS may vary depending on sex.

Albietz et al. [8] state that there is no correlation between ISS height at L4-L5 and age, in line with the findings of our study. On the other hand, there are studies that report that ISS distances in the lumbar region decrease with age [4, 16, 18]. This study found a negative correlation between the MHSS of L1-L2 and L2-L3 and age.

Sobottke et al. examined the correlation between ISS height and weight, height, and BMI. They reported that there was a positive correlation between ISS height and weight. This study found a negative correlation between AHISS at L3-L4 and weight. They also reported that there was a positive correlation between ISS height and the height of patients [4]. Our study found a positive correlation between PHISS and body size at all levels, and between MHISS and height at all levels except L3-L4. Additionally, Sobottke et al. reported that ISS height does not correlate with BMI [4]. Contrary to this, this study found a negative correlation between AHISS at L3-L4 and BMI.

Correlation of ISS height with age, weight, height, and BMI should be considered in order to manufacture correctly-sized interspinous devices, heighten the odds of success in their implantation and reduce possible complications.

In the previous studies, it was reported that the ISS height decreases from L1-L2 to L4-L5 [4, 14, 16, 18, 24]. This study found that while PHISS and MHISS decreased from L1-L2 to L4-L5, it was higher in AHISS at L2-L3 than at L1-L2, decreasing towards L4-L5 after L2-L3. Therefore, implantation and sizing of interspinous devices at the lower levels of the lumbar region need to be done more carefully.

While there are many studies on ISS height in the literature, there are only a few studies on LISS. In their study on L4-L5, Albietz et al. [8] stated that there was no correlation between LISS height at L4-L5 and age, in line with our study. This study found a positive correlation between LISS at L2-L3 and age. Moreover, this study found a positive correlation between LISS at L1-L2 and L2-L3 and weight, and a positive correlation between LISS and height at all levels. These correlations may provide useful information when designing and implanting interspinous devices.

The difference in the morphometric measurement results of SP and ISS in the literature may be due to the different reference points, measurement methods, number of images measured, and divergent imaging techniques. Furthermore, the inclusion of patients from different ethnicities, as well as variations in sex, age, height, weight, and BMI generate different results.

Limitations

The use of magnetic resonance images instead of computed tomography images for the measurements of spinous processes and interspinous spaces examined in this study can be considered as a limitation of this study. On the other hand, magnetic resonance images are known to be more reliable, especially in identifying soft tissues [27]. Since the ligamentum flavum is an important landmark in determining the parameters examined in this study, it was preferred to use magnetic resonance images.

## Conclusions

The intention of this study was to determine the reference intervals related to the morphometry of SP and ISSs at the lumbar levels. We found that at some levels, the height and length of the SP and ISS were greater for males than for females. We also examined how reference intervals depend on age, weight, height, and BMI, and observed statistically significant correlations at multiple levels. We think that these changes should be considered when designing and implanting interspinous devices. Since there are few studies examining all these correlations, we think that the results of this study will make a unique contribution to the literature.
